# The effects of MEX3A knockdown on proliferation, apoptosis and migration of osteosarcoma cells

**DOI:** 10.1186/s12935-021-01882-3

**Published:** 2021-04-08

**Authors:** Bangmin Wang, Zheping Hong, Chen Zhao, Qing Bi, Junhui Yuan, Jihang Chen, Yi Shen

**Affiliations:** 1 Department of The Affiliated, Cancer Hospital of Zhengzhou University, Jinshui District, No. 127 Dongming Road, Zhengzhou, Henan China; 2 Department of Orthopedics, Zhejiang Provincial People’s Hospital. No, Xiacheng District, 158 Shangtang Road, Hangzhou, Zhejiang China; 3 Department of Orthopedics, Furong District, The Second Xiangya Hospital of Central South University, No. 139 Middle Renmin Road, Changsha, Hunan China

**Keywords:** Osteosarcoma, MEX3A, Proliferation, Apoptosis, Cell cycle, Migration

## Abstract

**Background:**

Osteosarcoma is an aggressive malignant tumor which has attracted worldwide attention. MEX3A may be associated with tumors while has not yet seen its coverage on osteosarcoma. Herein, this study was to investigate the correlation between MEX3A and the progression of osteosarcoma.

**Methods:**

Firstly, we determined that expression of MEX3A was significantly higher in osteosarcoma tissues than that in marginal bone by immunohistochemical staining. Additionally, MEX3A expression was downregulated by the RNAi‐mediated knockdown. The functions of MEX3A knockdown on proliferation, apoptosis, cell cycle, migration was assessed by MTT assay, flow cytometry, wound-healing assay and Transwell assay, respectively. Knockdown of MEX3A resulted in suppressing cell proliferation, increasing cell apoptosis, inducing the G2 phase cell cycle arrest, and attenuating cellular migration. Furthermore, mouse xenograft model confirmed inhibitory effects of MEX3A knockdown on osteosarcoma formation.

**Results:**

The preliminary exploration on the molecular mechanism of MEX3A in osteosarcoma cells showed that the induction of apoptosis needs the participation of a series of apoptosis- associated factors, such as upregulation of Caspase 3, Caspase 8 and HSP60, downregulation of HSP27 and XIAP.

**Conclusions:**

In summary, these findings predicated that therapy directed at decreasing MEX3A expression is a potential osteosarcoma treatment.

**Supplementary Information:**

The online version contains supplementary material available at 10.1186/s12935-021-01882-3.

## Background

Osteosarcoma is the most common bone malignant tumor in children and young people, with highly invasive characteristics [1]. What is particularly terrible is that for osteosarcoma patients diagnosed with metastatic or recurrence, the 5-year survival rate is less than 30% [2]. The current treatment for osteosarcoma consists in neoadjuvant chemotherapy, surgery and postoperative chemotherapy [3–5]. The effort to improve the prognosis of osteosarcoma by intensifying multidisciplinary approach in patients with osteosarcoma has failed to show an improvement in survival rates, underscoring the urgent need for new treatment strategies [6]. Recently, with the development of osteosarcoma genomics, in-depth understanding found that the most common biological feature of the disease is genomic disorders, aneuploidy of chromosome alterations, deregulation of tumor suppressor genes and cell cycle, and lack of DNA repair [7–9]. Any disturbance in this process may contribute to aberrant differentiation and proliferation of these cells as well as malignant phenotype [7]. Therefore, these findings have significantly expanded the development of molecular targeted therapy strategy has gradually become a hot spot.

The *mex3* gene was identified for the first time in heterogeneous nuclear ribonucleoproteins, with a conserved region of 65–70 amino acids [10]. This region interacts with RNA and contains two K-homologous domains [11]. Existing evidence suggested that the MEX3 family is associated with epithelial homeostasis, embryonic development, metabolism, immune response, and cancer, but the specific mechanisms of these effects remain to be elucidated [12]. MEX3A, one of the four human gene families (MEX-3A, -3B, -3C, -3D) homologous to MEX3, is located on chromosome 1q22 and has 9986 base pairs [13]. Pereira et al., demonstrated that MEX3A was required for stemness in colon cancer cell lines [14]. Interestingly, MEX3A was identified as an independent prognostic factor of liver cancer [15]. Yet, the expression of MEX3A was not a poor prognostic factor of bladder urothelial carcinoma [16]. Furthermore, knockdown of MEX3A by small RNA interference suppressed cell proliferation and migration in human gastric cancer cells [17]. In addition, Krepischi et al., clarified that overexpression of MEX3A was associated with recurrence of Wilms tumor [18]. Recently, Wang et al., put forward the view that MEX3A downregulation inhibited the development of pancreatic ductal adenocarcinoma [19]. In view of the differences in the above results, the role of MEX3A in osteosarcoma aroused our curiosity.

In this study, we analyzed the expression of MEX3A in osteosarcoma and evaluated its potential role in osteosarcoma. Firstly, the difference of MEX3A expression level between osteosarcoma and marginal bone tissue was determined by immunohistochemical staining. Using in vitro experiments, we proved that the decreased expression of MEX3A has a certain inhibitory effect on the development and progression of osteosarcoma cells. Furthermore, mouse xenograft model confirmed the inhibitory effects of MEX3A knockdown on osteosarcoma formation. The preliminary exploration on the molecular mechanism of MEX3A in osteosarcoma cells showed that the induction of apoptosis needs the participation of a series of apoptosis-associated factors.

## Methods

### Microarray chips and immunohistochemical staining

A total of 104 microarray chips were purchased from Elena Biology (Cat. # OS804c), including 41 osteosarcoma tissue chips with TNM and clinical staging, and 11 marginal bone tissues. This study was approved by the Research Ethics Committee of Zhejiang Provincial People’s Hospital, and written informed consent was obtained from each participant.

Firstly, microarray chips were deparaffinized and blocked with citric acid antigen. Next, the chips were incubated with antibody MEX3A (1:200, abcam, Cat. # ab79046) at 4 °C overnight and then with HRP-conjugated goat anti-rabbit IgG (1:400, Beyotime, Cat. # A0208) for 2 h. Subsequently, they were stained first with DAB and then with hematoxylin. The staining of the tissues was observed with microscopic under 200× objective lenses. Pathological examination of microarray chips was carried out by two independent pathologists. In particular, the scores of IHC were determined by staining percentage scores [classified as: 1 (1–24%), 2 (25–49%), 3 (50–74%), 4 (75–100%)] and staining intensity scores (scored as 0: signal less color, 1: brown, 2: light yellow, 3: dark brown).

### Cell culture

The human osteosarcoma cells MNNG/HOS and U-2OS were purchased from the Cell Bank of the Chinese Academy of Sciences (Shanghai, China). They were cultured in essential medium MEM supplemented with 10% fetal bovine serum (FBS) (HyClone, Logan, UT, USA) and added 100 U/ml penicillin/streptomycin. Lentivirus-packed cells 293T were adherent epithelioid cells, and the growth medium was DMEM containing 10% FBS. All the cells were cultivated in a 37 °C incubator under the conditions of 5% CO_2_ and 95% humid air.

### Cell transfection, lentivirus production and infection

The small interfering RNAs specifically targeting (shMEX3A) sequences were designed to downregulation of MEX3A, and negative controls were scramble siRNAs (shCtrl). Sequences as follows: shMEX3A, 5′-AGGCAAGGCTGCAAGATTAAG-3′; shCtrl, 5′-TTCTCCGAACGTGTCACGT-3′. Synthesis of single-stranded DNA oligo containing interference sequences based on RNA interference target sequences, and annealing pairing to generate double-stranded DNA. Subsequently, it was directly ligated into the linear lentiviral vector BR-V-108, (5′-CCGGTTCTCCGAACGTGTCACGTTTCAAGAGAA-3′) (Shanghai Bioscienceres, Shanghai, China) through the restriction sites (Age I, NEB, Cat. # R3552L; EcoR I, NEB, Cat. # R3101L). The ligation product was transferred into competent cells *E. coli* TOP10 (TIANGEN, Cat. # CB104-03), and positive recombinants were identified by PCR. Three plasmids (Helper 1.0, Helper 2.0 and recombinant BR-V-108) were used to co-transfect 293T cells.

Lentivirus was collected 72 h after transfection. Next, various indicators of lentivirus were determined according to strict quality standards, such as physical index detection, sterility detection, and titer detection. Subsequently, MNNG/HOS and U-2OS at a density of 2 × 10^5^ cells/well for 24 h cultivation, which were infected with 100 μL recombinant lentiviral (1 × 10^7^ TU/mL) additive with green fluorescent protein (GFP) acted as a detectable marker. After 72 h, supernatants containing lentivirus expressing shCtrl and shMEX3A were harvested, respectively.

### RNA extraction and qPCR

Total RNA of MNNG/HOS and U-2OS was extracted with Trizol Reagent (Invitrogen, USA) according to manufacturer’s protocol. The RNA was used as a template for the production of cDNA with M-MLV RT kit (Promega). To analyze the mRNA expression of MEX3A, qPCR was performed with SYBR Green master mix (TaKaRa, Japan) on a BioRad CFX96 sequence detection system (Bio Rad Company, Berkeley, CA). The qPCR was calculated with relative quantification (2^−ΔΔCt^) method, which was normalized to GAPDH.

### Western blotting (WB) analysis

Total proteins from cells MNNG/HOS and U-2OS were extracted by whole cell lysis buffer and quantified using the bicinchoninic acid (BCA) method. The 20 μg of protein from each cell was electrophoresed by 10% SDS-PAGE and transferred to PVDF membranes. The PVDF membranes were sealed for 1 h with blocking solution (TBST solution containing 5% skim milk) at room temperature. After that, the membranes were incubated with a primary antibody MEX3A (1:1000, abcam, Cat. # ab79046) overnight at 4 °C and subsequently probed with secondary antibody HRP-conjugated goat anti-rabbit IgG (1:3000, Beyotime, Cat. # A0208). GAPDH (1:3000, Bioworld, Cat. # AP0063) served as a loading control. Immunoreactive blots were observed using ECL reagent (Pierce, Rockford, IL, USA), and band intensity was quantified using Image J software.

### MTT assay

Cells MNNG/HOS and U-2OS (2000/well) were seeded on 96-well plates. After incubation for 48 h, 20 μL of 5 mg/mL MTT (Genview, Cat. # JT343) was added to the medium, and cells were incubated at 37 °C for another 4 h. Then the culture medium was discarded and 100 μL dimethyl sulfoxide (DMSO) (Corning, Cat. # 10–013-CVR) was added to each well to dissolve the precipitate. Absorbance was measured at 490 nm using a microplate reader, with background subtraction measurements done at 570 nm. In the following 5 days, the absorbance of cell culture was measured every 24 h.

### Cell apoptosis analysis by flow cytometry

Cells MNNG/HOS and U-2OS were seeded in 6-well plates (2 ml/well) for 72 h cultivation and collected, washed twice with PBS and resuspended in 200 μL of 1 × binding buffer. Next, 10 μL Annexin V-APC was added for staining at room temperature and out of light for 15 min. Cell apoptosis rate was analyzed by flow cytometry (BD Bioscience, NJ, USA) and visualized in 3 randomly selected visual fields, which accessed by number of positive cells/numbers of all counted cells) × 100%.

### Cell cycle analysis by flow cytometry

Cells MNNG/HOS and U-2OS at a density of 2 × 10^3^ cells/well were plated in a 6-well culture plate (2 ml/well) and added 100 ng/mL nocodazole for 24 h to synchronize cells at the G1/S or G2/M boundary. Next, cells were trypsinized, washed twice with cold PBS and fixed with cold 70% ethanol at − 20 °C overnight. After that the cells were washed twice with PBS, incubated with 500 μL of 40 × propidium iodide (PI) (Sigma, Cat. # P4170) and 10 mg/mL RNase A for 30 min. The ratio of cells distribution in the G1, S and G2 phases was evaluated by flow cytometry.

### Wound-healing assay

Cells MNNG/HOS and U-2OS (50,000 cells/well) were seeded into 96-well plates (100 μL /well) and incubated for 24 h. Following, the cells overspread the bottom and were scratched by 10 μL pipette. PBS was used to wash out cell debris and suspension cells.

The next day, the low-concentration serum medium was changed, and the central part of the lower end of the 96-well plate was aligned with the scratch instrument, which was gently push upward to form a scratch. Serum-free medium was used for gently rinsing 2–3 times, then low concentration serum medium (e.g. 0.5% FBS) was added and photographed at 0 h, 4 h and 8 h at the same position.

### Transwell assay

Cells MNNG/HOS and U-2OS (80,000 cells/well) cells were cultured in the 24-well Transwell plates for 30 h. Following, 100 μL of serum-free medium containing 10^5^ cells was added into top chambers, and 600 μL DMEM containing 30% FBS was added into bottom chambers. After 24 h later, the non-metastatic cells in top chambers were removed by cotton swab. After fixation with 400 µL of 4% paraformaldehyde, the cells were stained with crystal violet staining solution and counted under microscope fields (200× magnification) per well.

### Mouse xenograft model

The animal procedures were conformed to protocols approved by the Animal Care Committee of Zhejiang Provincial People’s Hospital. The 4-week-old female BALB/c nude mice was purchased from Shanghai Slake laboratory animal company. MNNG/HOS cells (infected with shCtrl (n = 5) or shMEX3A (n = 5)) were injected into the back flanks of nude mice (n = 10) at a density of 4 × 10^6^ cells per 200 µL. After 15 days of subcutaneous injection, 0.7% pentobarbital sodium at a dose of 10 μL/g was injected into the abdominal cavity to anesthetize the mice and observe fluorescence using bioluminescence imaging (IVIS spectral imaging system, emission wavelength of 510 nm). Additionally, the tumor volume (tumor volume = π/6 × L × W × W, where L was long diameter and W was short diameter) were measured twice a week. The mice were sacrificed at day 41 after implantation, and the tumors were weighed and photographed.

### Ki67 staining

Mice tumor tissues were fixed in 10% formalin and then immersed in xylene and ethanol to dewax and rehydrate. To permeabilize the tissue, the sections were washed twice with 1% animal serum in PBS with 0.4% PBST. Next, they were incubated with anti-Ki67 (1:200, abcam, Cat. # ab16667) and HRP goat anti-rabbit IgG (1:400, abcam, Cat. # ab6721), respectively. Slides were stained by Hematoxylin (Cat. # BA4041, Baso) and then Eosin (Cat. # BA4022, Baso). The staining of the tissues was observed with microscopic under 200× objective lenses.

### Human apoptosis antibody array

Human Apoptosis Antibody Array kit (abcam, Cat. # ab134001) was performed to access the differential expression of apoptosis related proteins. Total proteins from MNNG/HOS cells were extracted by whole cell lysis buffer and quantified using the bicinchoninic acid (BCA) method. Following the manufacturer’s instructions, total proteins were incubated with blocked array antibody membrane overnight at 4 °C. After that, Detection Antibody Cocktail (1:100) was incubated at 4 °C for 2 h, followed with HRP linked streptavidin conjugate for 1 h. All spots were visualized by enhanced ECL and the signal densities were analyzed with ImageJ software (National Institute of Health).

### Statistical analysis

All experiments were performed in triplicate and data were presented as mean ± SDs. Statistical analyses and graphs were performed by GraphPad Prism 8.0 (Graphpad Software) and P value < 0.05 as statistically significant. The significance difference between groups were determined using the two-tailed Student’s t test or One-way ANOVA analysis.

## Results

### MEX3A was upregulated in osteosarcoma cells

**Fig. 1:**
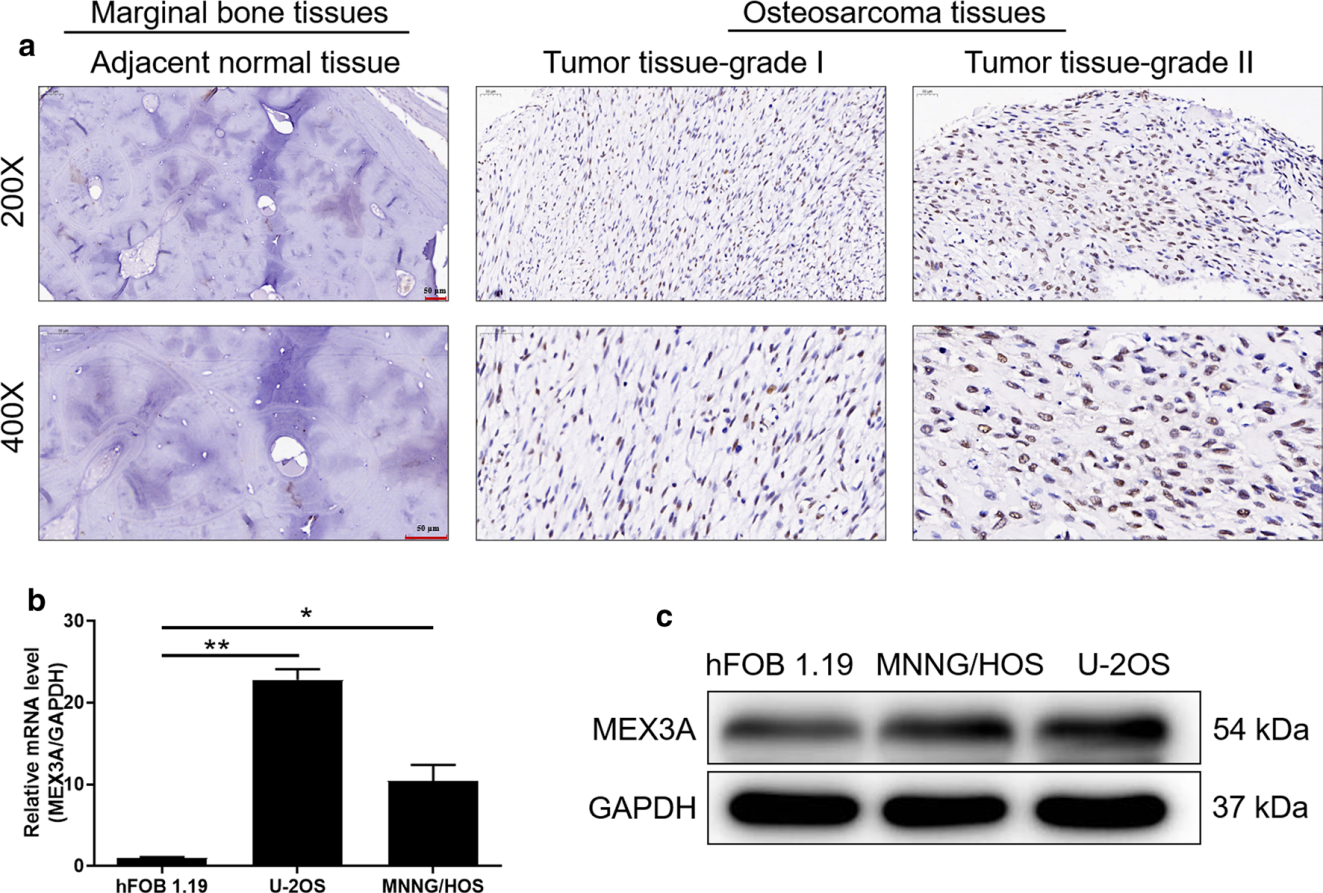
MEX3A is highly expressed in osteosarcoma. **a** Expression levels of MEX3A in osteosarcoma tumor tissues and adjacent normal tissues were detected by IHC staining (scale bar = 50 μm). **b**, **c** Expression levels of MEX3A in osteosarcoma cells were measured by qPCR (**b**) and WB (**c**). The data was presented as the mean ± SD (n = 3). *P < 0.05, **P < 0.01, ***P < 0.001

To evaluate expression of MEX3A in osteosarcoma, we analyzed 104 microarray chips of osteosarcoma patients with detailed clinicopathological information. For the statistical analysis, the chips were divided into two groups based on the median expression level of MEX3A: a low expression group (n = 22) and a high expression group (n = 82). As illustrated in Fig. 1a, the expression level of MEX3A in osteosarcoma tissues was markedly higher than that in adjacent normal tissues. On the other hand, the differential expression of MEX3A in osteosarcoma cell lines and control cell line hFOB 1.19 was detected (Fig. 1b, c), suggesting that MEX3A was highly expressed in MNNG/HOS and U-2OS. Additionally, to investigate the potential effect of MEX3A, we performed shRNA interference to decreased expression of MEX3A in MNNG/HOS and U-2OS cells. Fluorescence imaging was observed 72 h after transfection of MNNG/HOS and U-2OS with shCtrl and shMEX3A, and the results showed that the transfection efficiency reached over 70% (Additional file 1: Figure S1B). The interference efficiency of MEX3A was measured by qPCR, indicating that in MNNG/HOS and U-2OS was 96.1% (P < 0.05) and 94.94% (P < 0.01), compared with the shCtrl groups, respectively (Additional file 1: Figure S1B). As illustrated in Additional file 1: Figure S1D, the WB results showed that the protein level of MEX3A was decreased after shRNA-mediated knockdown. Accordingly, the cell models of downregulated MEX3A were successfully constructed in MNNG/HOS and U-2OS cells.

### Knockdown of MEX3A inhibited proliferation, promotes apoptosis in osteosarcoma cells

**Fig. 2:**
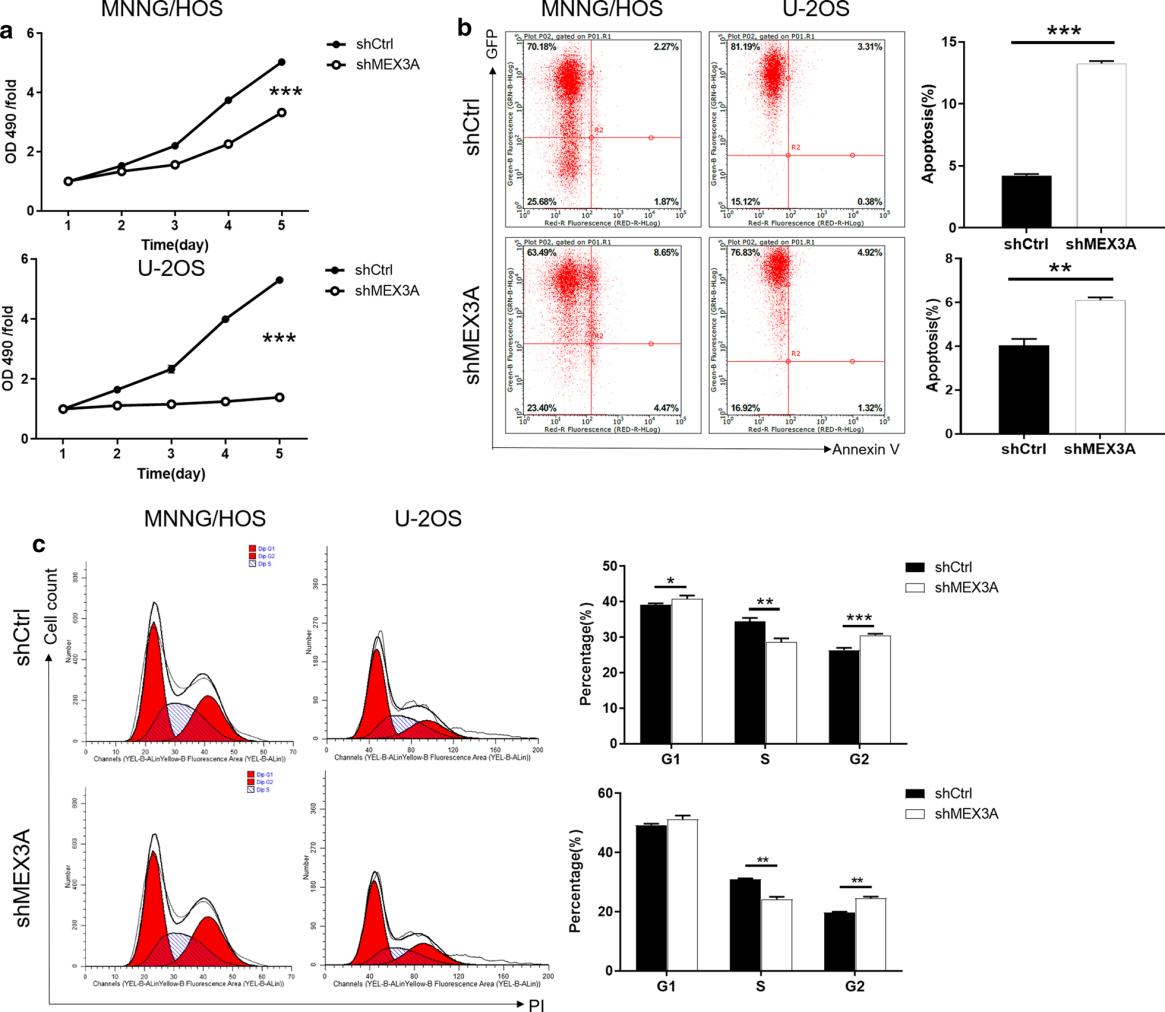
Knockdown of MEX3A inhibits cell proliferation, promotes apoptosis, arrests cell cycle of osteosarcoma cells. **a** Cell proliferation of MNNG/HOS and U-2OS cells with or without knockdown of MEX3A was evaluated by MTT assay. **b**, **c** Flow cytometry analysis based on Annexin V-APC staining was utilized to detect cell apoptotic ratio (**b**) and cell cycle distribution (**c**) for MNNG/HOS and U-2OScells. The representative images were selected from at least 3 independent experiments. The data was presented as mean ± SD (n = 3), *P < 0.05, **P < 0.01, ***P < 0.001

The comparison of the absorption rate of light at the wavelength of 490 nm between the shCtrl and shMEX3A over time was presented in Fig. 2a, where OD490 reflected the number of active cells. After 5 day of continuous detection, the results revealed that the OD 490/fold values of the shMEX3A group in MNNG/HOS and U-2OS cells were significantly lower than those of the shCtrl group (P < 0.001). In addition, apoptosis and cell cycle progression were detected by flow cytometry. On the one hand, the apoptosis rate of the shMEX3A group was higher than that of the shCtrl group in MNNG/HOS (P < 0.001) and U-2OS (P < 0.01) cells (Fig. 2b). On the other hand, the G2 phase cells numbers were increased in shMEX3A group compared to the shCtrl group (P < 0.01) (Fig. 2c). Taken together, these findings highlighted the role of MEX3A in osteosarcoma cells on proliferation, apoptosis and cell cycle.

### Knockdown of MEX3A suppressed the migration of osteosarcoma cells

**Fig. 3:**
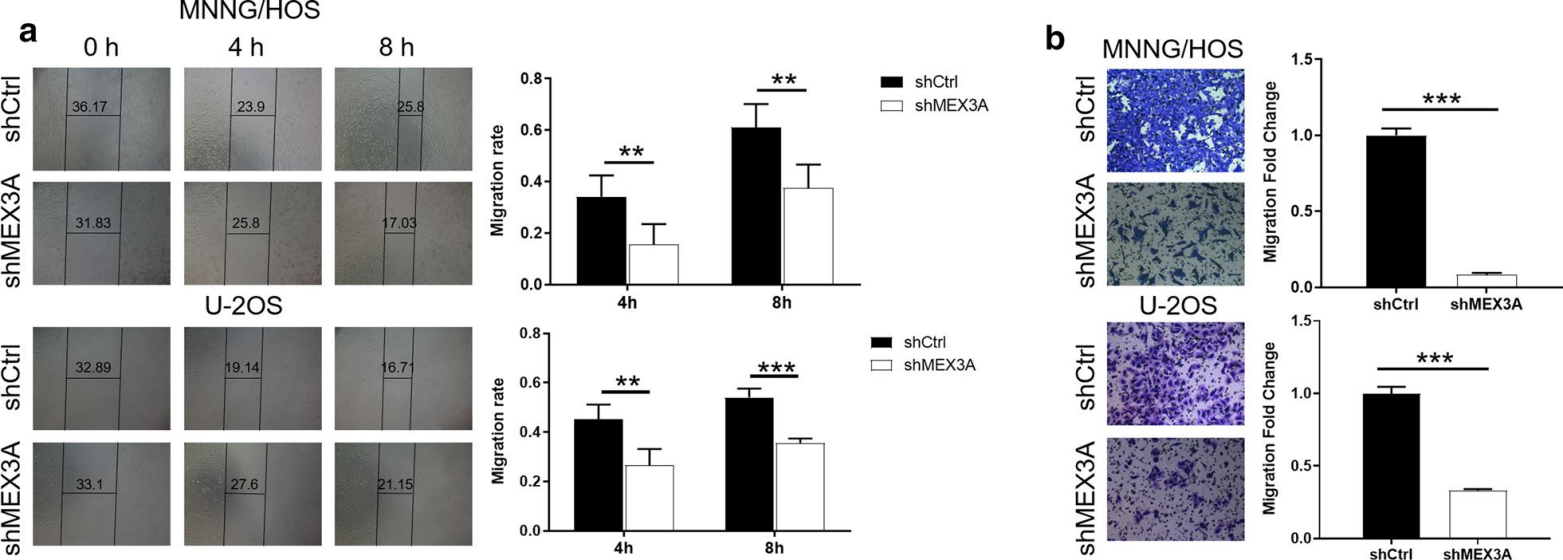
Knockdown of MEX3A inhibits cell migration of osteosarcoma cells. **a**, **b** Cell migration of MNNG/HOS and U-2OS cells with or without knockdown of MEX3A was evaluated by wound healing assay (**a**) and Transwell assay (magnification of 200×) (**b**). The representative images were selected from at least 3 independent experiments. The data was presented as mean ± SD (n = 3), *P < 0.05, **P < 0.01, ***P < 0.001

The wound healing assay and Transwell assay were used to measure cell migration. The shRNA-mediated knockdown of MEX3A in MNNG/HOS and U-2OS cells lead to sharply decreased migration ability, as indicated by a significant reduction in the migration rate during 8 h (Fig. 3a). Moreover, Transwell assay indicated that the number of MNNG/HOS and U-2OS cells in shMEX3A migrated to lower chamber decreased dramatically, suggesting that the downregulation of MEX3A reduced the migration ability of osteosarcoma cells (P < 0.001) (Fig. 3b). All in all, the inhibition of MEX3A reduced the migration ability of osteosarcoma cells.

### Knockdown of MEX3A inhibited growth of osteosarcoma cells in vivo

**Fig. 4:**
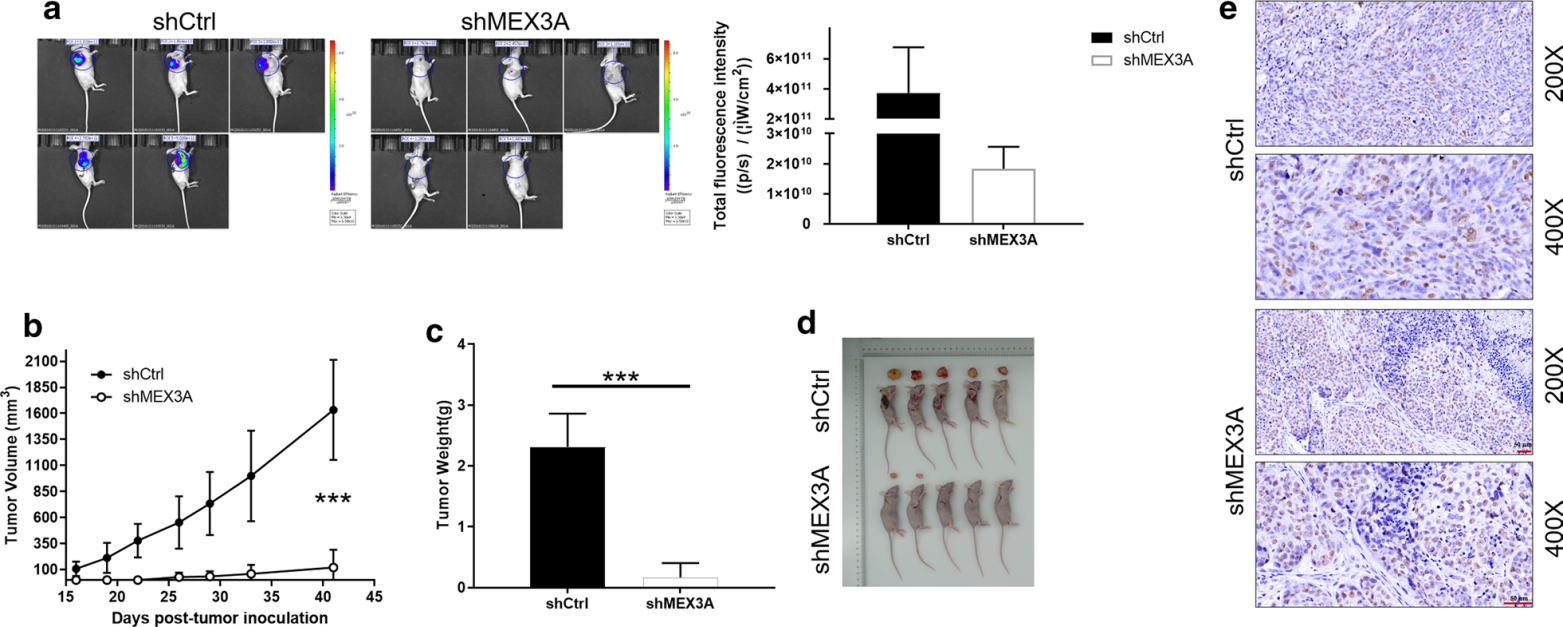
Knockdown of MEX3A inhibits osteosarcoma growth in mice xenograft models. **a** The fluorescence imaging and total fluorescence intensity of tumors in shCtrl group and shMEX3A group, respectively. **b** Images of mice and tumors in shCtrl group and shMEX3A group. **c** The volume of tumors in shCtrl group and shMEX3A group was measured after post-injection. **d** The average weight of tumors in shCtrl group and shMEX3A group. **e** Representative images of Ki67 staining in tumor tissue in the shCtrl and shMEX3A groups (scale bar = 50 μm). The data was presented as mean ± SD (n = 3), *P < 0.05, **P < 0.01, ***P < 0.001

Whether the downregulation of MEX3A can inhibit the growth of osteosarcoma cells in vivo remains to be further explored. The results of mouse xenotransplantation model were presented in Fig. 4. In vivo imaging showed that the total amount of fluorescence expression in the shMEX3A group was less than that in the control group, which indirectly reflected that the ability of tumor formation in the experiment was weakened (Fig. 4a). In addition, as illustrated in Fig. 4b, the tumor volume (Fig. 4c) and weight (Fig. 4d) of shMEX3A group were significantly lower than those of shCtrl group. More interestingly, Ki67 staining images (magnification: 200 ×) showed that the number of positive cells stained in shMEX3A group was significantly less than that in shCtrl group, which indicated that the downregulation of MEX3A resulted in a significant decrease in proliferation ability of osteosarcoma (Fig. 4e).

### Preliminary exploration on the molecular mechanism of MEX3A in osteosarcoma cells

**Fig. 5:**
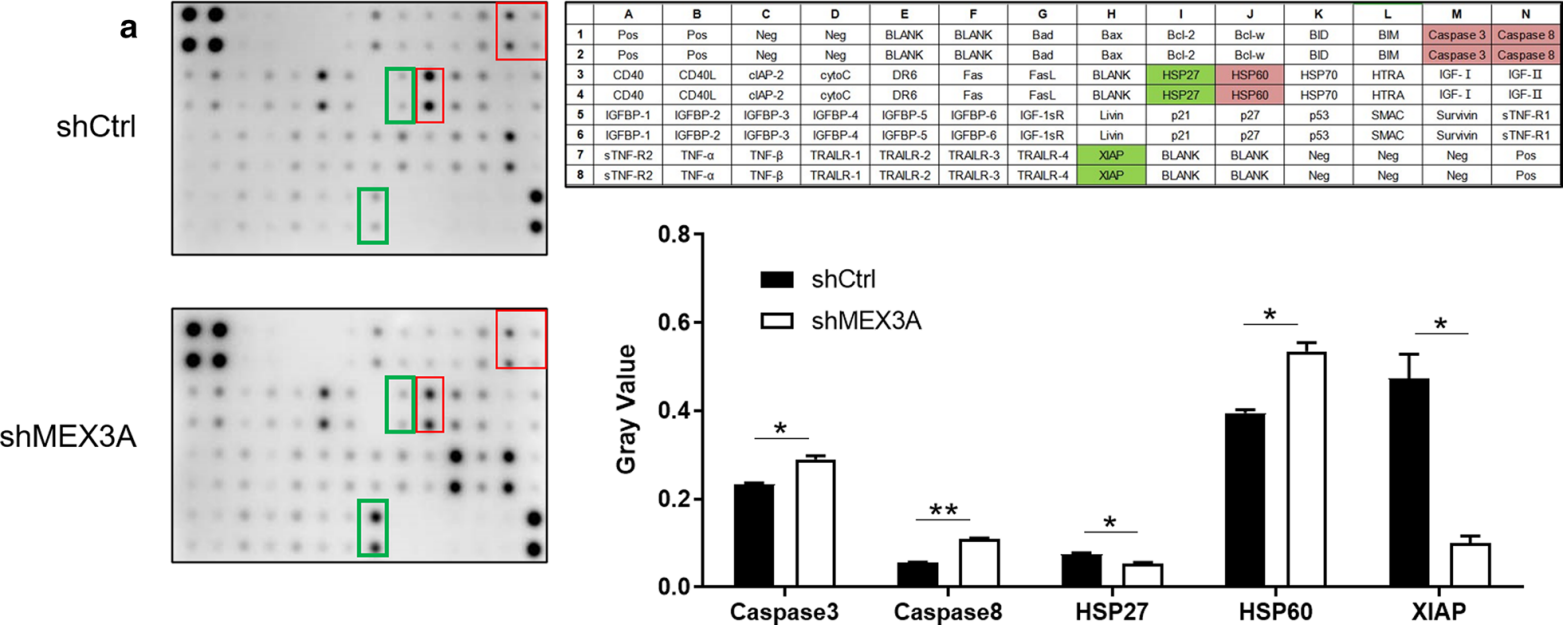
Exploration of downstream molecular mechanism of MEX3A in osteosarcoma cells. **a** Human apoptosis antibody array analysis was performed in MNNG/HOS cells with or without MEX3A knockdown; Densitometry analysis was performed and the gray values of differentially expressed proteins were shown. The data was presented as mean ± SD (n = 3), *P < 0.05, **P < 0.01, ***P < 0.001

The underlying mechanisms MEX3A of in osteosarcoma cells were investigated using a human apoptosis antibody array Kit. In MNNG/HOS cells, the related proteins of human apoptosis signal pathway were detected after RNA interfered with MEX3A. The differential expression of these apoptosis‐associated proteins between shCtrl and shMEX3A was shown in the Fig. 5a. In detail, the expression level of Caspase 3, Caspase 8 and HSP60 was significantly increased. On the contrary, the expression of HSP27 and XIAP was dramatically decreased (P < 0.05). In sum, knockdown of MEX3A could induce apoptosis in MNNG/HOS cells via regulating apoptosis‐associated proteins.

## Discussion

At present, a number of studies clearly illustrated that post-translational modification not only exerts a key role in controlling normal cells, but also in the initiation and progression of cancer [20, 21]. The RNA binding protein MEX3A was involved in the regulation of post-translational modification [22]. In this context, the role of MEX3A have garnered increasing attention in cancer with emerging evidence. In particular, the protein dysregulation of MEX3A was significantly associated with a variety of human malignant tumors, such as in gastric cancer, colorectal cancer, bladder cancer, and pancreatic ductal adenocarcinoma [14, 17, 23–25]. Besides, Bufalieri et al., reported that the MEX3A affects glioblastoma tumorigenesis by inducing ubiquitylation and degradation of RIG-I [26]. Albeit these, whether MEX3A was involved in the regulation of osteosarcoma cell function remained elusive. Here, we tried to illuminate the relation between MEX3A and osteosarcoma.

The current study determined that the expression of MEX3A was upregulated in osteosarcoma tissues than that in marginal bone. Additionally, in vitro results indicated that knockdown of MEX3A resulted in suppressing cell proliferation, increasing cell apoptosis, inducing the G2 phase cell cycle arrest and attenuating cellular migration. Furthermore, mouse xenograft model further supported inhibitory effects of MEX3A knockdown on osteosarcoma formation.

It was well known that the infinite proliferation, reduced apoptosis and cycle disorders are the hallmarks of cancers and exert an important role in its progression [27]. In addition, identification of apoptotic mechanisms was critical and facilitated the understanding of the diseases. Mechanisms of apoptosis and their effector proteins included pro-apoptotic protein, anti-apoptotic members, and inhibitor of apoptosis proteins [28]. Caspase-3, Caspase-8 and HSP60 were all pro-apoptotic proteins, which may contribute to apoptosis induction [29–31]. Kim et al., revealed that reversine induces Caspase-dependent apoptosis of human osteosarcoma cells through extrinsic and intrinsic apoptotic signaling pathways [32]. On the contrary, HSP27 could effectively blocked Caspase-dependent apoptotic pathways [33]. Moreover, Selvarajah et al., suggested that HSP60 expression was silenced using siRNA resulting in decreased cell proliferation and induction of apoptosis in osteosarcoma cells [34]. Qian et al., expounded that XIAP inhibitors could induce apoptosis leading to osteosarcoma cell death [35]. Consistently, we found that MEX3A knockdown induced apoptosis of osteosarcoma cells, resulting in the activation of Caspase-3, Caspase-8 and HSP60. Whereas, the expression of HSP27 and XIAP was dramatically suppressed. Therefore, MEX3A was involved in apoptosis induction of osteosarcoma cells requiring the participation of a series of apoptosis- associated factors.

## Conclusions

In conclusion, MEX3A was not only highly expressed in osteosarcoma tissues, but also involved in the regulation of the disease. These finding identified the role of MEX3A in osteosarcoma and drew further interest regarding its clinical utility as a potential therapeutic target.

## Supplementary Information

**Additional file 1: Figure S1.** The construction of MEX3A knockdown cell model.

## Data Availability

The data and analyzed during the current study are available from the corresponding author on reasonable request.
