# Supplementary material for: The effects of MEX3A knockdown on proliferation, apoptosis and migration of osteosarcoma cells
Source: Cancer Cell Int. 2021 Apr 8;21:197. doi: 10.1186/s12935-021-01882-3 (PMC8028067; doi:10.1186/s12935-021-01882-3)
Supplement: Supplementary file 1 — Additional file 1: Figure S1. The construction of MEX3A knockdown cell model. [file 12935_2021_1882_MOESM1_ESM.pdf]

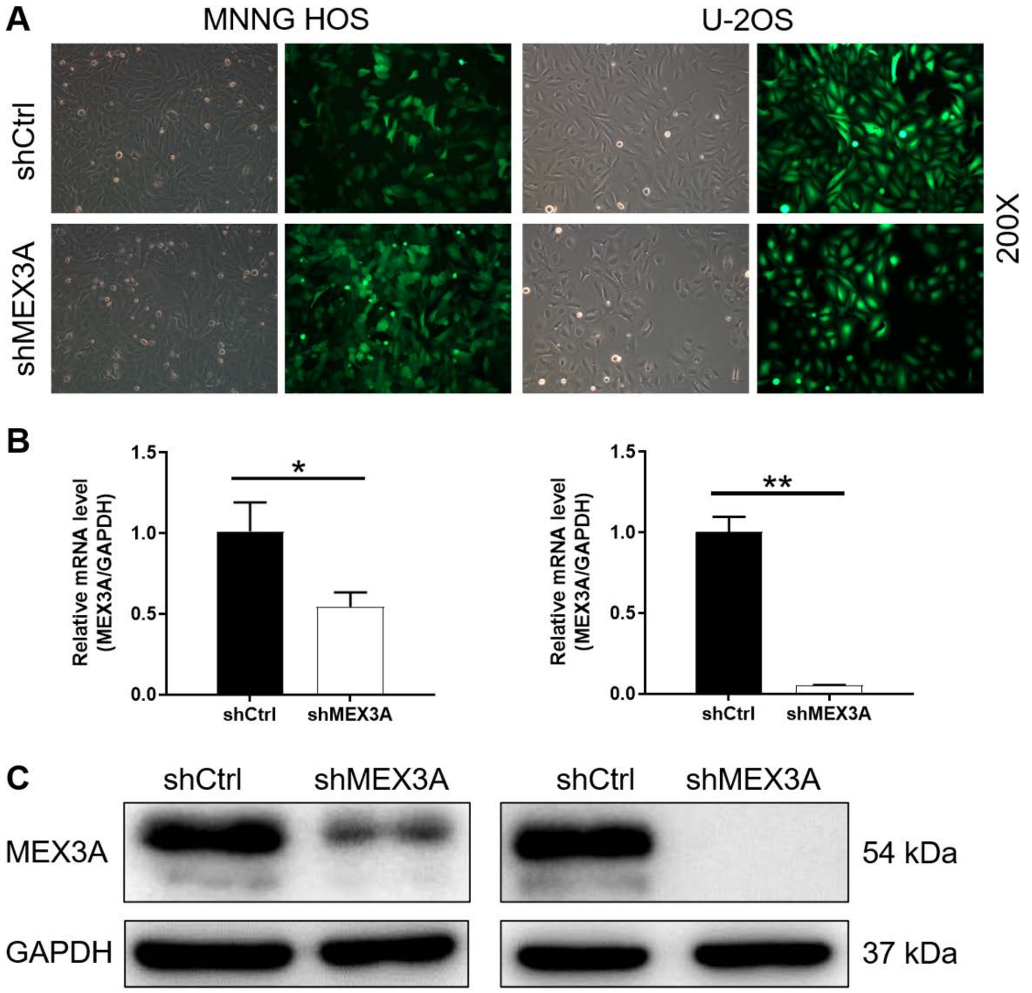

Figure S1. The construction of MEX3A knockdown cell model.

(A) Transfection efficiencies of MNNG/HOS and U-2OS cells were evaluated by expression of green fluorescent protein 72 h post-infection (magnification of 200×). (B, C) The specificity and validity of the lentivirus-mediated shRNA knockdown of MEX3A expression was verified by qPCR (B) and WB analysis (C). The data was presented as mean ± SD (n = 3), *P<0.05, **P<0.01, ***P<0.001.
